# Oxidative damage and antioxidant defense are assay and tissue‐dependent both in captive and wild‐caught bank voles (*Myodes glareolus*) before and after reproduction

**DOI:** 10.1002/ece3.4187

**Published:** 2018-07-06

**Authors:** Łukasz Ołdakowski, Jan R. E. Taylor

**Affiliations:** ^1^ Institute of Biology University of Białystok Białystok Poland

**Keywords:** antioxidative defense, bank vole, costs of reproduction, *Myodes glareolus*, oxidative damage, oxidative stress

## Abstract

Reproduction is costly and life‐history theory predicts that current parental investment will result in lower survival or decreased future reproduction. The physiological mechanisms mediating the link between reproduction and survival are still under debate and elevated oxidative damage during reproduction has been proposed as a plausible candidate. Previous studies of oxidative stress during reproduction in animals under natural conditions have been restricted to analyses of blood. Herein, we measured the level of oxidative damage to lipids (tiobarbituric‐acid‐reactive substances) and proteins (carbonyls) in the liver, kidneys, heart and skeletal muscles in free‐living bank vole females from spring and autumn generations, before and after reproduction. Antioxidant defense in the liver and kidneys was also determined. We expected oxidative damage to tissues and hypothesized that the damage would be more uniform between tissues in wild animals compared to those breeding under laboratory conditions. Considering all combinations of markers/tissues/generations, oxidative damage in females did not differ before and after reproduction in 12 comparisons, was lower after reproduction in three comparisons, and was higher after breeding in one comparison. The total glutathione was significantly increased after reproduction only in the liver of the autumn generation and there was no change in catalase activity. Our results confirm—for the first time in the field—previous observations from laboratory studies that there is no simple link between oxidative stress and reproduction and that patterns depend on the tissue and marker being studied. Overall, however, our study does not support the hypothesis that the cost of reproduction in bank voles is mediated by oxidative stress in these tissues.

## 1 INTRODUCTION

The trade‐off between reproduction and survival is a central concept of life‐history theory. Increased current investment in reproduction can decrease survival to the next reproduction event or decrease future reproductive success (Stearns, 1992). Throughout the breeding period, animals are faced with trade‐offs in the allocation of resources between self‐maintenance and reproductive activity because resources and/or their acquisition are limited (Roff & Fairbairn, 2007; Speakman, 2008; Stearns, 1992; Zera & Harshman, 2001). Even if resources are unlimited, animals may be constrained in their ability to expend energy (Hammond & Diamond, 1997; Peterson, Nagy, & Diamond, 1990; Speakman & Król, 2010), which would also force trade‐offs in energy utilization.

Of late, there has been considerable interest in the possibility that the trade‐off between reproduction and survival is mediated by oxidative stress: the imbalance between the production of reactive oxygen species (ROS) and the capacity of antioxidant mechanisms to neutralize the damaging effects of ROS. The investment in reproduction may reduce allocation of resources to somatic protection against ROS and repair mechanisms that would lead to oxidative damage to tissues. The idea that oxidative damage (to lipids, proteins, and DNA) is a consequence of reproductive effort has been extensively studied in recent years, but has received mixed support (Blount, Vitikainen, Stott, & Cant, 2016; Metcalfe & Monaghan, 2013; Monaghan, Metcalfe, & Torres, 2009; Selman, Blount, Nussey, & Speakman, 2012; Speakman & Garratt, 2013; Speakman et al., 2015). The general picture from studies on various species is very diverse: from an increase in the oxidative stress during reproduction (Alonso‐Alvarez et al., 2004; Bergeron et al., 2011; Fletcher et al., 2013) through the lack of significant differences between breeders and nonbreeders (Nussey, Pemberton, Pilkington, & Blount, 2009; Vitikainen et al., 2016), to the decrease in oxidative stress in breeding individuals (Costantini, Casasole, & Eens, 2014; Garratt, Pichaud, King, & Brooks, 2013; Garratt et al., 2011; Ołdakowski, Wasiluk, Sadowska, Koteja, & Taylor, 2015; Ołdakowski et al., 2012).

The majority of studies that have attempted to elucidate the role of oxidative stress in reproduction were carried out under controlled laboratory conditions, with ad libitum access to food, low thermoregulatory demands, and reduced physical activity. An important question is whether this situation adequately mimics the situation in the field with respect to the factors that generate oxidative stress. However, studies conducted on wild animals in their natural environment, especially on mammals, that provide important information about their real costs of living, are still scarce (Bergeron et al., 2011; Christensen et al., 2015; Fletcher et al., 2013; Nussey et al., 2009; Sharick, Vazquez‐Medina, Ortiz, & Crocker, 2015).

In recent years, it has become clear from multiple studies of laboratory mammals that different tissues respond in different ways to the imposition of reproduction (Al Jothery et al., 2016; Ołdakowski et al., 2012, 2015; Vitikainen et al., 2016; Xu, Yang, Speakman, & Wang, 2014; Yang, Xu, Wang, & Speakman, 2013). Studies in the field, however, have been exclusively restricted to analyses of a single tissue (blood) [see the above references for mammals; Speakman and Garratt (2013) for references to seven species of birds; Isaksson, While, Olsson, Komdeur, and Wapstra (2011) and Wilson, Gravel, Mackie, Willmore, and Cooke (2012) for reptiles and the fish]. It is therefore unclear whether the diversity in tissue response is an artifact of studying animals in captivity with ad libitum access to food that is generally also supplemented with large amounts of exogenous antioxidants. Such dietary antioxidants might, for example, explain the widespread observation of reduced oxidative damage in the liver of reproducing rodents (but see Vaanholt et al., 2016). It might be hypothesized that under field constraints there may be a more uniform tissue‐wide response.

**Figure 1:**
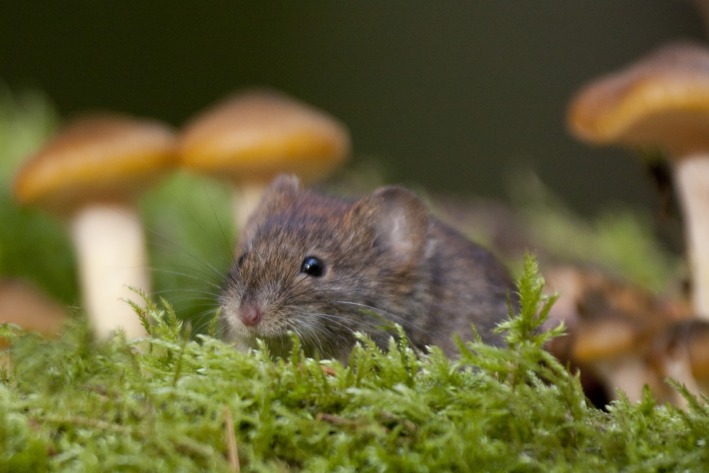
Research subject: the bank vole *Myodes glareolus*. Photograph credits: Karol Zub

We sought to test this hypothesis in this study. Until that time, we have demonstrated in captivity that the effect of reproduction varies between tissues in the bank vole (*Myodes glareolus* Schreber, 1780; Figure 1) (Ołdakowski et al., 2012, 2015). Bank voles are small rodents that are abundant in European forests. They can be easily adapted to breeding in laboratory conditions (Sadowska, Baliga‐Klimczyk, Chrząścik, & Koteja, 2008). In the first experiment (Ołdakowski et al., 2012), bank vole females were allowed to produce one or two litters, and used females without access to males as a control group. In the second experiment (Ołdakowski et al., 2015), we manipulated the reproductive effort of bank vole females by either increasing or decreasing litter size in four consecutive litters and nonbreeding females were controls. These laboratory studies did not reveal oxidative stress in breeding bank voles; levels of oxidative damage were unchanged in some tissues or were reduced in others.

**Figure 2:**
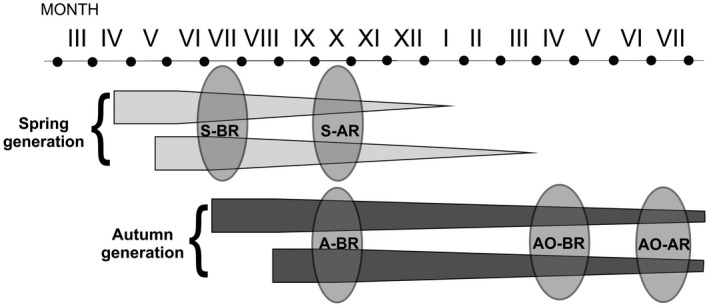
Timing of trapping of bank vole females (depicted with ovals). S‐BR = Animals from the spring generation, before reproduction, S‐AR = spring generation, after reproduction, A‐BR = females from the autumn generation, before reproduction, AO‐BR = overwintered animals, mainly individuals from the autumn generation of the previous year, before reproduction, AO‐AR = overwintered animals, after reproduction. The age structure of bank vole populations after Gliwicz (1983)

In this study, we measured the level of oxidative damage to lipids and to proteins, and the level of antioxidants (the activity of catalase and the concentration of total glutathione) in different tissues of free‐living bank vole females collected before or after reproduction. The set of tissues examined was the same as in our laboratory study. Bank voles give birth to young between late April and the end of September and therefore two generations of young, spring and autumn generations, can be distinguished (Gliwicz, 1983; Figure 2). Voles from the spring generation reproduce in the same calendar year and those from the autumn generation at an older age, after overwintering. We included animals from both generations in our study. We expected that if the natural habitat is a more stressful environment than that under laboratory conditions, then reproduction would result in oxidative damage to tissues of free‐living voles. In other words, we expected higher oxidative damage in females after reproduction than before. Measuring the oxidative damage soon after reproduction, the cumulated effect of past reproductive investment is of great interest from the life‐history point of view because lasting damage may have consequences for future performance and survival. We tested the hypothesis that the damage would be more uniform between tissues—and would not show the same diversity of response observed in our laboratory study and those reported from laboratories by other authors. We also hypothesized that increased damage would be associated with reduced antioxidant protection.

## 2 MATERIALS AND METHODS

### 2.1 Animals

Bank vole females belonged to 5 groups according to their reproductive status (before or after reproduction) and age (Table 1, Figure 2). They were trapped in Knyszyn Forest near Czarna Białostocka, NE Poland, in autumn 2012, and spring and summer of 2013. Wooden box traps used for trapping were open from around 2 PM until approximately midnight and checked every two hours. Upon capture, voles were sexed and females without visible signs of pregnancy or lactation were taken to a field laboratory where they were weighed to nearest 0.1 g (on the Ohaus Scout SC 4010 balance) and killed by cervical dislocation. The liver, kidneys, heart (drained of blood), and skeletal muscles associated with the femur were dissected, weighed (± 0.001 g; Radwag WPA 71 balance) and immediately snap‐frozen in liquid nitrogen. The frozen tissues were stored at −80°C until analysis.

**Table 1:** Characteristics of the five groups of bank vole females under study

Group	N	Generation	Before or after reproduction	Age	Trapping season
S‐BR	10	Spring	Before	Young of the year	Early summer
S‐AR	6	Spring	After	Young of the year	Autumn
A‐BR	11	Autumn	Before	Young of the year	Autumn
AO‐BR	10	Autumn	Before	Overwintered	Early spring
AO‐AR	6	Autumn	After	Overwintered	Early summer

N, number of individuals.

Reproductive status (before reproduction or after reproduction) was determined through examination of placental scars (visible or not) in the uterus (Alibhai, 1982; Nyholm & Meurling, 1979). Age class was assigned according to the root length of the first lower molar (Tupikova, Sidorova, & Konovalova, 1968; Wasilewski, 1952); for this measurement, the tooth was removed from the mandible after soaking it in papain solution (Pankakoski & Hanski, 1989; Searle, 1954). Body mass of females and trapping season served as additional information for age determination.

### 2.2 Analyses of oxidative damage and antioxidants

All assays were described in detail in Ołdakowski et al., 2015;. In brief, tissues were homogenized in cold 50 mmol/L phosphate buffer with 1 mmol/L EDTA and pH 7.0. The dilution of tissues in the homogenate was 1:10 w/v in the assays of the oxidative damage to liver and in determinations of catalase activity, and 1:15 w/v in all other assays. The supernatant was obtained by centrifugation at 10,000 *g* for 15 min at 4°C and kept on ice to determine oxidative damage. To quantify the lipid peroxidation in tissues, the level of malondialdehyde (MDA) was determined spectrophotometrically after reaction with thiobarbituric acid (TBA) by the modified method of Ohkawa (Ohkawa, Ohishi, & Yagi, 1979). Protein content in each sample was determined by the Lowry method with Peterson modification (Lowry, Rosebrough, Farr, & Randall, 1951; Peterson, 1977) and the concentration of TBARS (thiobarbituric acid reactive substances) was expressed in nmol/mg protein. The damage to proteins was quantified by measuring protein carbonyls by spectrophotometry after reaction with 2,4‐dinitrophenylhydrazine (DNPH) (Levine, Williams, Stadtman, & Shacter, 1994; Stadtman & Oliver, 1991). The protein content was determined in control tubes by measuring absorbance, and final protein carbonyl concentration was expressed in nmol/mg protein.

Catalase activity in the liver and kidneys was determined according to Aebi's (1986) method and was expressed as the rate constant of a first‐order reaction (k) per mg protein. The total glutathione concentration in liver was measured according to Anderson (1996) along with modification for use with a 96‐well plate reader (Vasilaki et al., 2006) and expressed in nmol per mg protein. In both analyses, protein content was measured with the Lowry method with Peterson modification (Lowry et al., 1951; Peterson, 1977).

Although the tissue samples were collected in different seasons of the year, all determinations were performed at the same time to avoid random effects. Repeatability (the intra‐class correlation coefficient; Lessels & Boag, 1987) of the assays of oxidative damage and antioxidants was higher than 0.90.

### 2.3 Statistical analyses

All statistical tests were performed in SAS (version 9.3; SAS Institute, Cary, NC, USA). We tested differences in body mass, levels of oxidative damage and antioxidants before and after reproduction in females from spring and autumn generations. In the spring generation, we used a *t* test to compare females before (S‐BR) and after reproduction (S‐AR; see Table 1 for abbreviations). To compare TBARS in muscles in these two groups, the *t* test for unequal variances was used. In the autumn generation, three means were analyzed by means of ANOVA (general linear model): young of the year trapped in autumn (A‐BR), overwintered females trapped in early spring, still before reproduction (AO‐BR), and females after reproduction (AO‐AR). For comparison of A‐BR with AO‐AR and AO‐BR with AO‐AR, we used pairwise planned contrasts. To meet parametric assumptions, the damage to lipids in muscles was transformed logarithmically. Because, we tested the differences in four organs/tissues, we applied the step‐down Bonferroni procedure for adjusting significance levels to control type I error rates. We did not include body mass in the models; body mass was statistically significant only in the comparison of TBARS in kidneys and carbonyls in the heart in the spring generation and correcting for body mass did not change the outcome of the tests. Pearson correlation coefficient was used to check possible relationships between the oxidative damage (to lipids and proteins) and the antioxidant defense (the activity of catalase and the concentration of the total glutathione) in a pooled sample of all 43 collected females.

All experimental procedures were accepted by the Local Ethical Committee in Białystok (permission 29/2012).

## 3 RESULTS

**Figure 3:**
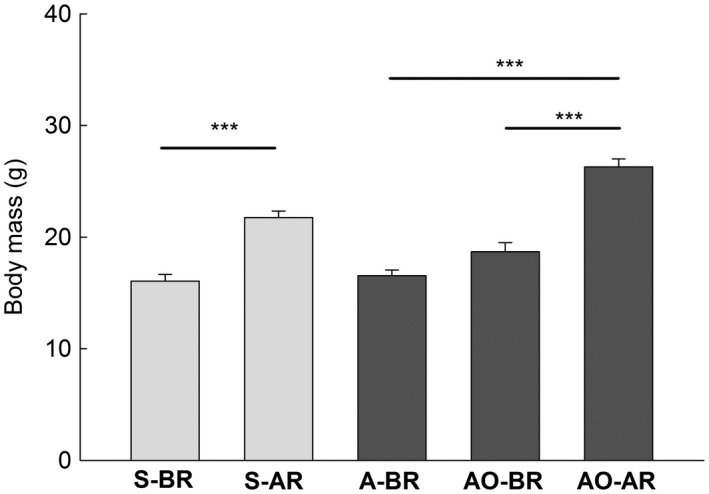
Mean body masses (g, +*SE*) of bank vole females in different groups. See Table 1 for abbreviations of group names and characteristics. The lines above bars show compared pairs of groups and statistical differences between groups (see *Statistical analyses*): ns = nonsignificant; * = *p *<* *0.05; ** = *p *<* *0.01; *** = *p *<* *0.001

**Figure 4:**
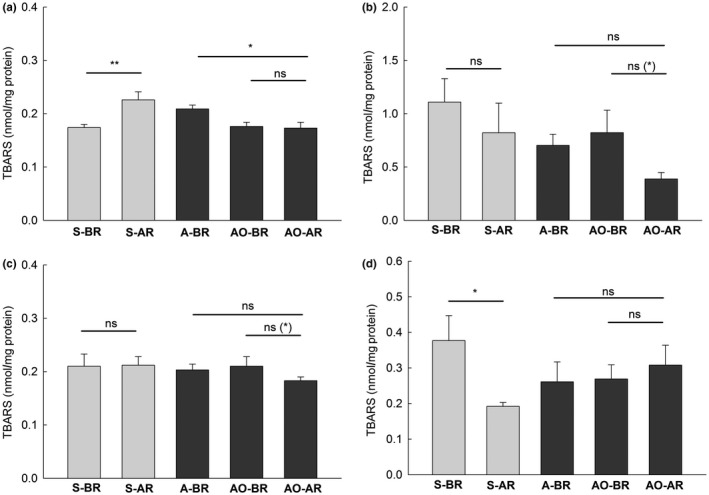
Mean concentrations (+*SE*) of TBARS (thiobarbituric acid reactive substances), a marker of oxidative damage to lipids in the liver (a), kidneys (b), the heart (c), and skeletal muscles (d) of bank vole females in different groups and the differences between groups after applying the Bonferroni adjustment. See Table 1 and Figure 3 for other explanations. The significance level of the difference between means without the Bonferroni adjustment is shown in parentheses above the horizontal lines if different from the level after applying the adjustment

**Figure 5:**
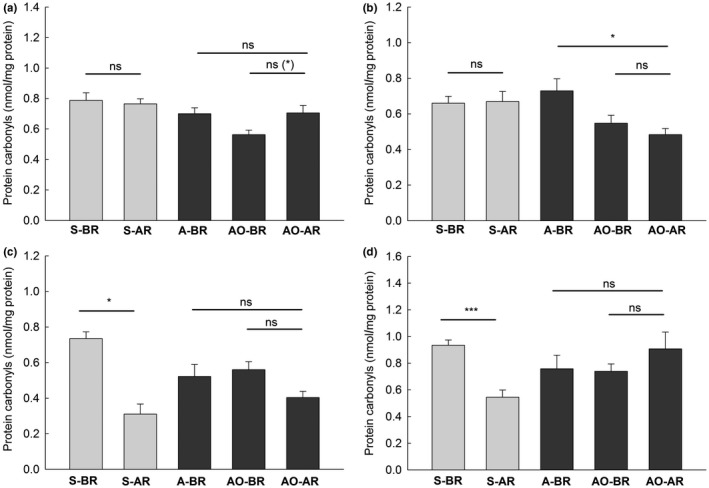
Mean concentrations (+*SE*) of protein carbonyls, a marker of oxidative damage to proteins, in the liver (a), kidneys (b), the heart (c), and skeletal muscles (d) of bank voles in different groups. See Table 1 and Figure 3 and 4 for other explanations

In both generations, females after reproduction were heavier than females before reproduction (Figure 3). Comparison of the oxidative damage to tissues after and before reproduction (S‐AR *vs*. S‐BR and AO‐AR *vs*. AO‐BR) provided varied results. The concentration of TBARS, markers of oxidative damage to lipids, was significantly higher in females after reproduction than in females before reproduction, only in the liver in the spring generation (Figure 4a). However, in the other three comparisons, the change in the damage to tissues with breeding was in the opposite direction. The level of TBARS in skeletal muscles and the concentration of carbonyls in the heart and muscles were lower after reproduction in animals from the spring generation Figures 4d, 5c,d). The differences in the remaining 12 comparisons of the marker/tissue combinations were nonsignificant (Figures 4 and 5). Because the Bonferroni procedure has been criticized (Moran, 2003), we also report nonadjusted significance levels in Figures 4 and 5. The concentration of markers was higher, lower, and did not differ after reproduction in 2, 5, and 9 comparisons, respectively, when Bonferroni correction was not applied.

The comparison of the autumn generation females, those after reproduction (AO‐AR) and those in the autumn of a previous year (A‐BR), showed a lower concentration of TBARS in the liver and carbonyls in the kidneys after reproduction. The differences in the remaining six comparisons of the marker/tissue combinations were nonsignificant (Figures 4 and 5). There were no differences between the Bonferroni adjusted and nonadjusted significance levels in these comparisons (Figures 4 and 5).

**Figure 6:**
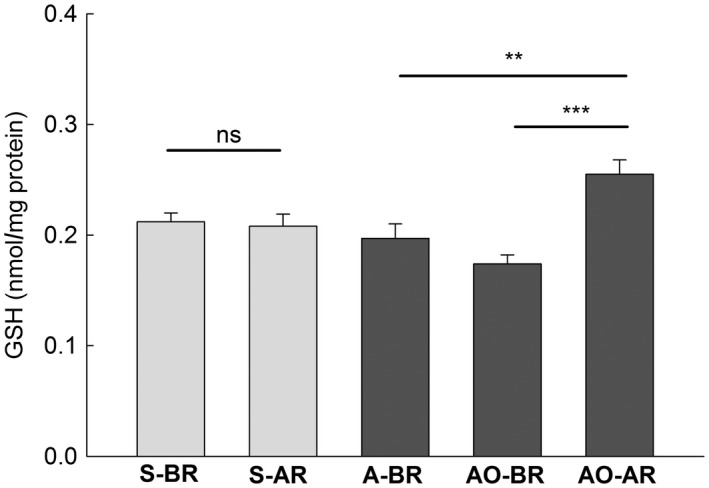
Mean concentrations (+*SE*) of the total glutathione (GSH) in the liver of bank voles in different groups. See Table 1 and Figure 3 for other explanations

The level of total glutathione in the liver was significantly increased after reproduction in the autumn generation (AO‐AR group compared with AO‐BR and A‐BR groups; Figure 6; Table 2) but in the spring generation, the difference was nonsignificant. The activity of catalase before and after reproduction did not differ either in the liver or kidneys (Table 2).

**Table 2:** Comparison of antioxidants: catalase activity and the total glutathione (GSH) concentration in bank vole females after and before reproduction

Organ	Antioxidant	Spring generation		Autumn generation			
		S‐AR *vs*. S‐BR		AO‐AR *vs*. A‐BR		AO‐AR *vs*. AO‐BR	
		*t*_14_	*p*	*F*_1,24_	*p*	*F*_1,24_	*p*
Liver	Catalase	1.98	0.067	0.05	0.824	0.54	0.470
	GSH	0.28	0.785	10.18	**0.0039**	19.20	**0.0002**
Kidneys	Catalase	1.20	0.250	0.76	0.391	1.64	0.212

See Table 1 for abbreviations.

*p*‐values of statistically significant effects are shown in bold.

We tested whether the level of oxidative damage in the liver and kidneys was related to antioxidant defense. The level of the total glutathione was significantly positively correlated with the concentration of protein carbonyls in the liver but all other correlations between the antioxidants and the markers of damage were nonsignificant (Table 3).

**Table 3:** Pearson coefficients of correlation between the markers of oxidative damage to lipids (TBARS) and to proteins (protein carbonyls) and the antioxidant defense (catalase and the total glutathione, GSH) in the liver and kidneys

Organ		TBARS		Protein carbonyls	
		*r*	*p*	*r*	*p*
Liver	Catalase	−0.197	0.206	−0.067	0.657
	GSH	0.115	0.462	0.332	**0.029**
Kidneys	Catalase	−0.279	0.070	−0.088	0.575

*p*‐value of the statistically significant correlation is shown in bold.

## 4 DISCUSSION

### 4.1 Oxidative damage to tissues before and after reproduction

Oxidative stress has been suggested as a proximate cost of reproduction (Metcalfe & Monaghan, 2013; Monaghan et al., 2009; Selman et al., 2012). Expectations based on life‐history theory predict that investment in antioxidant defenses should decline during reproduction, leading to elevated oxidative damage. Hence, considering that natural habitat is a potentially more stressful environment than the laboratory, we expected increased oxidative damage to tissues in bank vole females trapped at the end of the breeding season and more uniform response of different tissues than in our laboratory study. This expectation was additionally supported by the fact that our vole females of both generations increased their body mass during breeding (Figure 3), and the intense growth could also generate oxidative stress (Nussey et al., 2009).

Contrary to our predictions, our study revealed very mixed responses of various tissues to reproduction. Moreover, the direction of the changes was in some cases opposite to the expectation. In the majority of the comparisons, females after reproduction did not differ from females just before reproduction in the level of oxidative damage to tissues. In three tissue/marker comparisons, the level after reproduction was significantly lower. Only the level of lipid peroxidation in the liver in the spring generation was enhanced in females after reproduction (Figure 4 and 5). No sign of oxidative damage due to reproduction was found in females from the autumn generation when females from autumn of the previous year were used as a reference; the damage was lower after reproduction in two comparisons, and no difference was found in the remaining six. We treat these eight comparisons as complementary in inferring about oxidative stress because they include winter period that preceded breeding.

In general, the analysis of oxidative damage in four tissues did not support the oxidative stress associated with reproduction in bank voles. This conclusion holds true also when the Bonferroni correction to the probability values is not applied (Figures 4 and 5).

Nevertheless, it cannot be excluded that assays in other tissues would reveal significant oxidative damage. An important point is that no signs of oxidative stress were found in females from two very different generations, born in different seasons of the year.

The results of the present study on voles trapped in the field are in line with our earlier laboratory experiments that concerned the role of oxidative stress in reproduction in the same species and involved the same tissues and markers. Females which weaned two litters had unaltered oxidative damage or lower damage (TBARS in kidneys and muscles) in relation to nonbreeding females (Ołdakowski et al., 2012). In another study, where females were forced to wean four consecutive manipulated (either enlarged or decreased) litters, these two groups of breeding females did not differ and had lower damage levels (TBARS in the liver, carbonyls in the liver and the heart) or unaltered levels of oxidative damage compared with nonreproducing females (Ołdakowski et al., 2015). In addition, females selected for high maximum aerobic metabolic rates (which also had high resting metabolic rates and food intake) did not differ in oxidative damage from nonselected females. These results together cast doubt on the presence of oxidative stress in breeding bank vole females, at least in the tissues which were analyzed.

The response of four tissues to reproduction was completely different in three studies on the bank vole, including the present one (Ołdakowski et al., 2012, 2015; this study). In the present study, the response was different even between the spring and autumn generations. This shows that the effects of reproductive status on oxidative stress are critically dependent on the markers and tissues used, not only in laboratory studies (as was previously shown by Xu et al., 2014; Yang et al., 2013; Al Jothery et al., 2016) but also in animals in the wild. Although the cause of this variation is not known, it is clear that if we had only measured one tissue or used one marker, we might reach opposite conclusions regarding oxidative stress depending on the tissue we had chosen.

Our study adds to a mixed picture of the role of oxidative stress in reproduction under natural conditions. We are aware of only five studies that have examined oxidative stress in breeding free‐living mammals and all of them reported oxidative damage to blood plasma. Eastern chipmunk (*Tamias striatus*) females showed a weak positive effect of litter size on the TBARS level (Bergeron et al., 2011). Females of Soay sheep (*Ovies aries*) which produced two surviving offspring in the previous spring, had a lower concentration of TBARS than females with one or no surviving offspring (Nussey et al., 2009). In contrast, in another cross‐sectional study, oxidative damage to proteins in lactating female North American red squirrels (*Tamiasciurus hudsonicus*) exceeded by 1.9 times that in nonbreeding females (Fletcher et al., 2013). Female northern elephant seals (*Mirounga angustirostris*), which undergo prolonged fasts during breeding, showed increases in oxidative damage to proteins (Sharick et al., 2015). However, in another longitudinal study, banded mongoose (*Mungos mungo*) females showed reduced levels of TBARS during pregnancy and the level of TBARS after reproduction did not differ from that before breeding (Vitikainen et al., 2016). The latter study supports the “oxidative shielding” hypothesis, which suggests that mothers attempt to shield their offspring from oxidative damage during pregnancy (Blount et al., 2016). Overall, these five studies provided very mixed results on the role of oxidative stress in reproduction. However, they show that a cross‐sectional study does not necessarily exclude the demonstration of the costs (Fletcher et al., 2013) and a longitudinal study does not always demonstrate such costs (Vitikainen et al., 2016).

### 4.2 Oxidative damage vs antioxidant defense

We found a significant positive correlation between protein carbonyls and the level of total glutathione (GSH) in the liver. All other correlations between the markers of the oxidative damage and the antioxidants (GSH and catalase) were nonsignificant (Table 3). Significantly increased GSH in the liver after reproduction in the autumn generation (Figure 6) could play a protective role and could result in the observed lack of increased damage to lipids and proteins during reproduction in these females (characteristically, the lack of increase of GSH after reproduction in the spring generation females correlated with the increase of the damage to lipids; Figures 4a and 6). Five of six correlations between the oxidative damage and the antioxidant defense were nonsignificant (Table 3). This, combined with the lack of significant differences in the activity of catalase between groups of voles, is consistent with the conclusion of no oxidative stress in the tissues under study.

### 4.3 Study design limitations

In this study, the cross‐sectional analysis was applied; the oxidative damage was not determined in the same individuals before and after reproduction, but we sampled different individuals from the population. We are aware of all disadvantages of this approach. The most serious drawback is that animals, which survived and were captured might have a low level of the oxidative damage and animals with high levels of oxidative damage might simply not have survived and were eliminated from the population, resulting in the underestimated oxidative stress after reproduction. We also cannot exclude the possibility that high‐quality females, who invested much in reproduction (for example those who could defend territory better and were able to start to breed earlier), were characterized by relatively low oxidative stress after breeding, that might obscure the association between reproduction and oxidative damage. A longitudinal study would require taking blood samples from the same individual before and after reproduction to enable determination of the potential oxidative damage to the blood. However, aside from problems with bleeding in small rodents, we were interested in oxidative damage to the liver, the heart, kidneys, and muscles, the same tissues that we studied in our laboratory experiments on bank vole females (Ołdakowski et al., 2012, 2015); these determinations could be made only once. The consequences of the damage to different tissues for survival are not known. However, it was reported that in two rodent species during reproduction, superoxide dismutase was downregulated in the blood, paralleling the increased damage, and upregulated in the liver, paralleling the reduced damage (Plumel et al., 2014; Xu et al., 2014; Yang et al., 2013). This may suggest that during reproduction, females may differentially allocate protection between different tissues (Speakman & Garratt, 2013). It may be speculated that protection of “solid” tissues is more important for animal performance than of blood plasma because the turnover of plasma constituents is high.

## 5 CONCLUSIONS

All previous studies of oxidative stress during reproduction in animals under natural conditions have reported levels of damage only to blood. The present study is the first in the wild to report oxidative damage to other tissues. Contrary to our expectations, we did not find any clear signs of oxidative stress associated with reproduction in the tissues under study. Our hypothesis that the damage would be more uniform among tissues in voles collected in the wild (as compared with laboratory studies) was also not supported; the effect of reproduction varied between tissues, markers, and generations. The cause of this variation is not clear and there is an urgent need to elucidate whether damage to any tissue results in decreased survival of parents and their future reproductive potential.

## CONFLICT OF INTEREST

None declared.

## AUTHORS’ CONTRIBUTIONS

JRET conceived the ideas; JRET and ŁO designed methodology; ŁO collected the data; ŁO and JRET analyzed the data and wrote the manuscript. All authors contributed critically to the drafts and gave final approval for publication.

## DATA ACCESSIBILITY

Data available from the Dryad Digital Repository: https://doi.org/10.5061/dryad.779k1h4
